# Prostate Cancer Postoperative Nomogram Scores and Obesity

**DOI:** 10.1371/journal.pone.0017382

**Published:** 2011-02-24

**Authors:** Jacqueline M. Major, Hillary S. Klonoff-Cohen, John P. Pierce, Donald J. Slymen, Sidney L. Saltzstein, Caroline A. Macera, Dan Mercola, Michael W. Kattan

**Affiliations:** 1 Division of Cancer Epidemiology and Genetics, National Cancer Institute, National Institute of Health, Bethesda, Maryland, United States of America; 2 Department of Family and Preventive Medicine, School of Medicine, University of California San Diego, La Jolla, California, United States of America; 3 UCSD Moores Cancer Center, La Jolla, California, United States of America; 4 Department of Epidemiology and Biostatistics, Graduate School of Public Health, San Diego State University, San Diego, California, United States of America; 5 Department of Pathology and Laboratory Medicine, University of California Irvine, Irvine, California, United States of America; 6 Department of Quantitative Health Sciences, Cleveland Clinic Foundation, Cleveland, Ohio, United States of America; Florida International University, United States of America

## Abstract

**Purpose:**

Nomograms are tools used in clinical practice to predict cancer outcomes and to help make decisions regarding management of disease. Since its conception, utility of the prostate cancer nomogram has more than tripled. Limited information is available on the relation between the nomograms' predicted probabilities and obesity. The purpose of this study was to examine whether the predictions from a validated postoperative prostate cancer nomogram were associated with obesity.

**Methods:**

We carried out a cross-sectional analysis of 1220 patients who underwent radical prostatectomy (RP) in southern California from 2000 to 2008. Progression-free probabilities (PFPs) were ascertained from the 10-year Kattan postoperative nomogram. Multivariable logistic regression models estimated odds ratios (ORs) and 95% confidence intervals (CIs).

**Results:**

In the present study, aggressive prostate cancer (Gleason ≥7), but not advanced stage, was associated with obesity (p = 0.01). After adjusting for age, black race, family history of prostate cancer and current smoking, an inverse association was observed for 10-year progression-free predictions (OR = 0.50; 95% CI = 0.28–0.90) and positive associations were observed for preoperative PSA levels (OR = 1.23; 95% CI = 1.01–1.50) and Gleason >7 (OR = 1.45; 95% CI = 1.11–1.90).

**Conclusion:**

Obese RP patients were more likely to have lower PFP values than non-obese patients, suggesting a higher risk of experiencing prostate cancer progression. Identifying men with potentially higher risks due to obesity may improve disease prognosis and treatment decision-making.

## Introduction

Obesity constitutes a growing public health problem that may influence the outcome of a number of chronic diseases, including cancer. In the U.S., the prevalence of obesity has increased dramatically since 1980, a trend that has been observed across age and ethnic subgroups. In 2000, approximately 65% of adults were overweight and 30% were obese [1], [2].

Carcinoma of the prostate is the most frequently diagnosed non-skin cancer and the 2^nd^ leading cause of cancer deaths in males in the U.S. In 2009, there were 192,280 incident cases and 27,360 estimated deaths from prostate cancer [3], [4]. Several lines of research suggest that lifestyle factors may be involved in progression of prostate cancer and development of potentially fatal disease [5], [6]. Clinicopathological characteristics determined at time of surgery (pre-operative serum PSA) or immediately postoperatively (e.g. stage, grade, margin status) have constituted a particularly important area of focus because of their ability to prognosticate recurrence. These isolated disease characteristics have not been found to be consistently associated with body mass across studies [7], [8], [9], [10], [11], [12]. The prostate cancer nomogram, a composite measure that incorporates a group of clinicopathologic characteristics, is used by both clinicians and patients at the time of diagnosis [13]. Since its conception in 2004, the utility of the prostate cancer nomogram has more than tripled. Yet, limited information is available on the potential associations between lifestyle factors, including obesity, with the nomograms predicted probabilities. Such associations would be useful in further characterizing high-risk prostate cancer patients [14]. The purpose of this study was to examine the association of obesity with the updated 10-year postoperative prostate cancer nomogram and its individual components.

## Materials and Methods

### Population

The Strategic Partners for the Evaluation of Cancer Signatures (SPECS) is an ongoing observational study that uses specimen tissues and clinical data to derive gene signatures for the prognosis of prostate cancer at the time of diagnosis. Men were eligible for study recruitment if they had been diagnosed as having prostate cancer, scheduled to undergo a radical prostatectomy, and didn't have prior radiation or hormonal therapy for prostate cancer. Men were recruited during the pre-operation clinic visit. Subjects for the present study consisted of 1298 biopsy-confirmed prostate cancer patients who underwent prostatectomy between 2000 and 2008 at four centers in southern California. Of the 1298 participants, subjects with missing data for clinicopathologic factors required for the 10-year postoperative nomogram were excluded from the analyses (n = 78, overlap exists). The final study population for the present analysis consisted of 1220 subjects. Signed informed consent was obtained for all participants. Institutional review board approval for the present study was obtained from the University of California Irvine, University of California San Diego, and San Diego State University.

### Measures and Procedures

Demographics and anthropometrics were ascertained at the preoperative clinic visit. Date of birth, race (White, Black, Asian, Hispanic, Other), current smoking status (yes/no), family history of prostate cancer (yes/no) were assessed by self-administered questionnaires. Body mass index (BMI) was calculated by dividing the patient's weight in kilograms by their height in meters squared (kg/m^2^). Obesity was defined as BMI ≥30 according to the WHO international classification. Pathologic review and reporting was performed according to standards described by the TNM classification [15]. Preoperative PSA level was abstracted from the medical record and pathologic characteristics were obtained by examination of pathology reports by trained clinical study coordinators. Surgical margins were categorized as positive or negative, positive if tumor was present at the inked specimen surface. Pathological Gleason sum was divided into two groups according to histology: well differentiated (≤6) and poorly differentiated (≥7). Pathological stage was categorized as organ-confined (T1/T2) or not organ-confined (T3/T4) disease.

Progression-free probabilities (PFP), the probability of avoiding disease progression, e.g., biochemical recurrence, were derived using the 10-year postoperative Kattan nomogram (http://www.mskcc.org/applications/nomograms/Prostate/PostRadicalProstatectomy.aspx). Details of the postoperative Kattan nomogram have been described [16]. Briefly, the nomogram is a robust predictive tool which incorporates preoperative PSA level, year of surgery, Gleason grade (primary and secondary), surgical margins, pathologic stage and lymph node involvement to predict the 10-year probability that a prostate cancer will not progress after RP. The nomogram has been validated in independent samples with predictive accuracy (Harrell's concordance index: 0.79 to 0.81; area under the curve (AUC): 0.89).

### Statistical analyses

Descriptive statistics were tabulated for patient characteristics. Univariate analysis was performed with chi-square tests for categorical variables and Wilcoxon rank sum for continuous variables.

Associations of obesity with the 10-year postoperative PFP (and each of its individual clinicopathologic components) were performed using multivariable logistic regression. Serial models were used to assess potential confounding. All models adjusted for age, given the documented associations of age with both BMI and prostate cancer. The final model adjusted additionally for black race, positive family history and current smoking. Odds ratios (OR) and corresponding 95% confidence intervals (CI) were calculated. Tests of interaction by age, black race, family history and current smoking were performed. For analysis, PSA levels were log-transformed to normalize the data. PFP values were transformed using the arcsine-square root transformation. After observing a significant association between obesity and PFP, post hoc analyses were performed to determine which of the individual components comprising the PFP index was associated with obesity.

Data were analyzed using SAS® (version 9.1, SAS Institute Inc., Cary, NC) and Hmisc package in R software (version 2.8). All p-values were based on two-tailed tests of significance.

## Results

The characteristics of the 1220 patients analyzed in the present study are shown in Table 1. Mean age at time of surgery was 62 years (range 40 to 80) and the study population was predominantly white, with only 5% of black descent. Twenty-three percent reported having a family history of prostate cancer. Mean BMI was 27.7 kg/m^2^ (range: 18.3 to 48.8). In our study population, 305 (25%) men were identified with BMI ≥30 kg/m^2^. Preoperative PSA levels ranged from 0.1 to 78.2 ng/mL with a median value of 5.8 ng/mL (95% CI = 4.3–8.5). Fifty-seven percent of the subjects had a Gleason sum ≥7 and 10% had values ≥8. With regards to tumor stage, the majority (approximately 77%) had disease confined to the prostate defined as T1/T2; however, 21% had cancer that had extended beyond the prostatic capsule or into the seminal vesicles (T3) and 16 men had cancer that spread to the bladder (T4). About 27% of the subjects who underwent RP had positive margins. The proportions of subjects with extra-capsular extension, seminal vesicle invasion and/or positive lymph nodes were small (19%, 8% and 3%, respectively), consistent with reports in other RP patient populations.

**Table 1:** Characteristics of patients undergoing radical prostatectomy.

Characteristic	Men (N = 1220)	
Age (years), mean (SD)	61.9	(7.0)
Race/ethnicity, n (%)		
White	892	(73.1)
Asian	156	(12.8)
Hispanic	63	(5.2)
Black	62	(5.1)
Other	47	(3.8)
Family history of prostate cancer, n (%)	278	(22.8)
Current smoker, n (%)	159	(13.0)
Weight (kg), mean (SD)	87.3	(14.1)
Height (cm), mean (SD)	177.5	(7.8)
BMI (kg/m^2^), mean (SD)	27.7	(4.1)
Preoperative PSA, median (95% CI)	5.8	(4.3–8.5)
Pathologic Gleason sum, n (%)		
3–6	524	(43.0)
7 (3+4)	431	(35.3)
7 (4+3)	139	(11.4)
8–10	126	(10.3)
Pathologic stage, n (%)		
T1	7	(0.6)
T2	939	(77.2)
T3	255	(20.9)
T4	16	(1.3)
Positive margins, n (%)	324	(26.6)
Extra-capsular extension, n (%)	232	(19.0)
Seminal vesicle invasion, n (%)	98	(8.0)
Node positive, n (%)	40	(3.3)

BMI, body mass index; PSA, prostate-specific antigen; SD, standard deviation.

Unadjusted associations between PFP scores and obesity are reported in Table 2 along with comparisons for the individual clinicopathologic characteristics. Gleason sum was strongly associated with obesity in the univariate analysis (*P* = 0.03), particularly for Gleason ≥7 (63% vs. 55%, *P* = 0.01) as was the predicted progression-free probabilities from the 10-year postoperative nomogram (*P* = 0.02). The median values of preoperative PSA were slightly higher in obese subjects (6.0 vs. 5.7, *P* = 0.05), albeit not a clinically meaningful difference. The proportion of subjects with preoperative PSA levels ≥10 was higher (22% vs. 17%) in obese men compared to non-obese men. Further, a larger proportion of obese subjects had positive surgical margins (30% vs. 25%); however, these differences were not statistically significant (*P* = 0.07). No differences were observed between the two groups for the remaining individual pathological characteristics.

**Table 2:** Clinical and pathologic tumor characteristics according to obesity.

	BMI<30		BMI ≥30		
Characteristic	No.	%	No.	%	*P* value
No. of patients	915		305		
Preoperative PSA, ng/mL					
Median (95% CI)	5.7 (4.2–8.4)		6.0 (4.5–9.2)		.05†
≥10 ng/mL	159	17.4	67	22.0	.07
Pathologic Gleason sum					.03
3–6	412	45.1	112	36.7	
7 (3+4)	319	34.6	112	36.7	
7 (4+3)	99	11.1	40	13.1	
8–10	85	9.2	40	13.4	
≥7	503	55.0	193	63.3	.01
Pathologic stage					.34
T1	5	0.5	2	0.7	
T2	710	77.8	229	75.3	
T3	189	20.7	66	21.7	
T4	9	1.0	7	2.3	
T3/T4	198	21.7	73	24.0	.40
Positive margins	231	25.3	93	30.5	.07
Extra-capsular extension	177	19.3	55	18.0	.61
Seminal vesicle invasion	71	7.8	27	8.8	.54
Node positive	30	3.3	10	3.3	.89
10-y Postoperative PFP, %					
Mean (SD)	89 (0.2)		87 (0.2)		.02‡

BMI, body mass index; PSA, prostate-specific antigen; PFP, progression-free probability.

*P* value based on χ^2^ test unless otherwise indicated.

† Wilcoxon rank sum test;

‡ Student's t-test.

**Figure 1:**
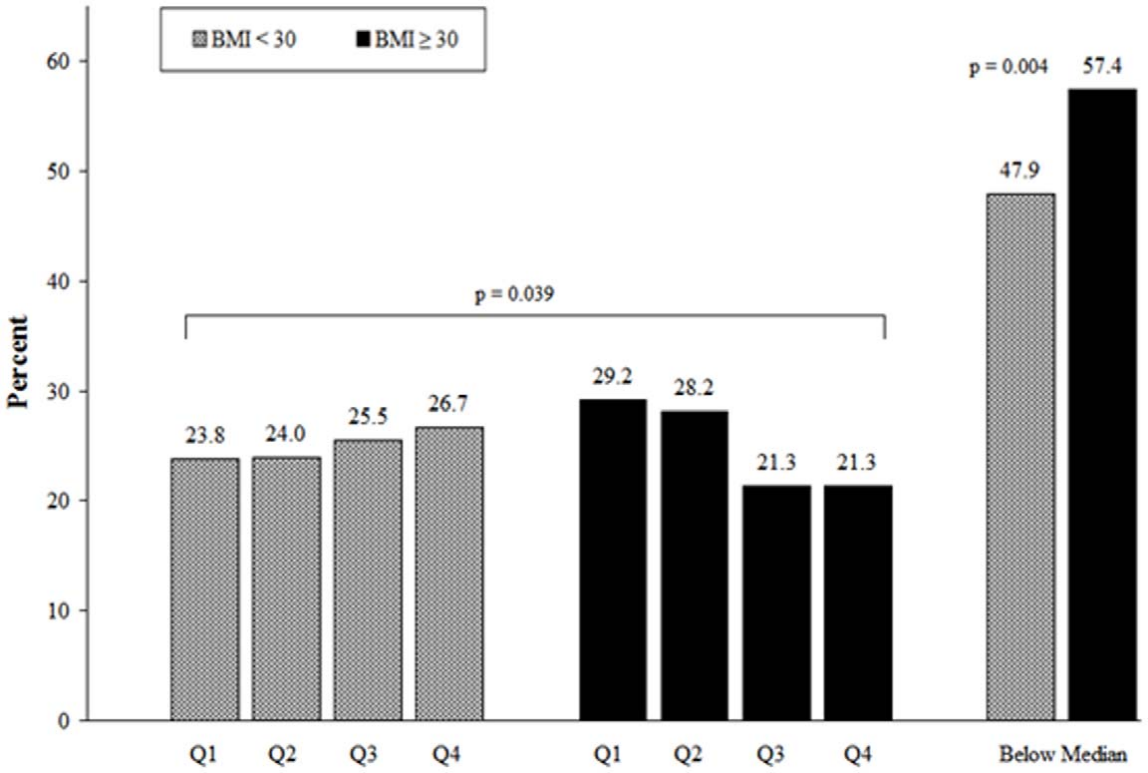
Distribution of PFP quartiles by obesity.

Patients were divided into quartiles based on postoperative nomogram predicted 10-year PFP (Figure 1). We observed a significant difference between the obese and non-obese subjects (*P* = 0.039). A larger proportion of obese subjects had PFP values in the lower two quartiles (Q1 and Q2). Within the non-obese men, only marginal differences were shown in the proportion of subjects within each PFP quartile (range 23.8 to 26.7%). The difference between obese and non-obese subjects becomes more apparent when we dichotomize PFP values into two groups, below and above the median. The proportion of obese subjects who have PFP values below the median (i.e., a worse prediction) is significantly higher than the proportion of non-obese subjects (57.4 vs. 47.9, *P<*0.01).

Associations between obesity and the 10-year postoperative nomogram as well as its individual components are reported in Table 3. In age-adjusted logistic regression analyses, men with a pathologic Gleason sum ≥7 were 1.44 times more likely to be obese than those with a Gleason sum <7 (OR = 1.44, 95% CI = 1.10–1.88) and the odds of being obese increased by a factor of 1.24 for every 1-ng/mL increase in PSA levels. The result for preoperative PSA was only marginally significant (95% CI = 1.02–1.50). In the fully-adjusted model, these associations did not change markedly. No associations were found between obesity and pathologic stage, surgical margins, extra-capsular extension (ECE), seminal vesicle invasion (SVI) and lymph node (LN) involvement.

**Table 3:** Adjusted associations between obesity* and RP patient characteristics.

	Model 1		Model 2	
Characteristic	OR	95% CI	OR	95% CI
Black race	1.59	0.90–2.78		
Positive family history	0.87	0.64–1.19		
Current smoker	0.92	0.62–1.36		
Preoperative PSA† (ng/mL)	1.24	1.02–1.50	1.23	1.01–1.50
Pathologic Gleason sum, ≥7	1.44	1.10–1.88	1.45	1.11–1.90
Pathologic stage, T3/T4	1.16	0.86–1.58	1.18	0.86–1.60
Positive margins	1.30	0.98–1.73	1.29	0.97–1.72
Extra-capsular extension	0.93	0.66–1.30	0.94	0.67–1.32
Seminal vesicle invasion	1.19	0.75–1.89	1.20	0.75–1.92
Node positive	0.96	0.45–2.05	0.99	0.48–2.07
10-y postoperative PFP‡				
1% increase	0.51	0.28–0.91	0.50	0.28–0.90
20% increase	0.87	0.78–0.98	0.87	0.77–0.98

OR, odds ratio; CI, confidence interval; PFP, progression-free probability.

*Obesity was defined as BMI ≥30;

† Postoperative PSA was log-transformed;

‡ PFP was arcsine-transformed.

Model 1: adjusting for age.

Model 2: adjusting for age, black race, family history, current smoker.

A significant inverse association was observed between obesity and the PFP nomogram predictions. The odds of being obese decreases by a factor of 0.87 for every 20% increase in PFP (OR = 0.87, 95% CI = 0.77 = 0.98), after adjusting for age, black race, family history and current smoking. Based on this finding, obese RP patients in our study were more likely to have lower PFP values, suggesting a higher risk of experiencing prostate cancer progression.

## Discussion

In the first study to examine the relation between obesity and the 10-year postoperative prostate cancer nomogram, our findings suggest an independent inverse association between obesity and the probability of remaining progression-free.

Our findings are in accord with those from the one study, to our knowledge, that examined the association of body mass and predicted PFP from the 7-year postoperative nomogram [11]. In this study, researchers examined data from 702 men with a mean age of 59 years who had undergone RP from 1988 to 2006 and found that obese patients were predicted on average to have an absolute decrease in their probability of remaining free of progression (74.3% vs. 80.1% for obese and non-obese men, respectively; p = 0.04). A limitation noted by the authors is that they used the 7-year rather than the 10-year Kattan nomogram. By incorporating year of surgery, the 10-year postoperative nomogram provides more accurate predictions by taking into account advances in screening over time [16], [17], [18]. Because PFP was not the main focus of their analysis, comparison of PFP values between obese and non-obese men was performed without adjusting for potential confounding factors that might have been important given the reported differences in mean age and year of surgery between the obese and non-obese men in their study population. We were able to confirm their findings when using the 10-year nomogram and adjusting for potential confounders.

In two separate cohorts of RP patients with clinically localized disease, researchers conclude that body mass index (BMI) does not improve the predictive accuracy of statistical models beyond that which is already explained by clinicopathologic factors. However, preoperative and postoperative factors were not examined simultaneously and potential confounders, such as age and year of surgery, were not accounted for in the analyses. Each study modeled progression within a short period of follow-up (median follow-up of 25.9 months) [19], [20].

Body mass may be linked to lower PFP scores and poorer disease pathology through a variety of adipose tissue induced-hormonal changes (e.g., increased levels of insulin and bioavailable IGF-I that are known to have mitogenic properties). Most proposed mechanisms point to markers of aggressiveness or extent of disease spread. Our study suggests evidence to support the former (e.g., Gleason) but not the latter; we did not observe associations with extent of disease as measured by pathologic stage, ECE, SVI, or LN involvement. One possible explanation may be that TNM stage, unlike Gleason grade, is determined by both the rate of disease growth (a feature of the degree of aggressive potential of the disease) and time of detection. Therefore, observed associations between obesity and stage may be reflective of the influence of obesity on the timing of detection. Conversely, Gleason grade is more purely a reflection of the innate aggressiveness of the disease and is largely unaffected by the timing of detection. Given this, we might expect that a true biological effect of obesity on prostate cancer would more likely register as an association with grade rather than stage.

The present study has both strengths and limitations. A major strength is the detailed assessment of pathologic tumor characteristics and objective assessment of weight and height prior to surgery [21], which is particularly important given that weight after surgery may not be a good measure due to changes as a result of disease, treatment, or lifestyle changes. We employed the updated version of the postoperative nomogram, a validated prediction tool, which incorporates year of surgery. Additional strengths include the availability of information that allowed us to adjust for risk factors that may play a role in prostate cancer progression such as age, race, family history and cigarette smoking [3], [22], [23], [24], [25], [26], [27].

Limitations of the present study include a one-time measure of BMI. In addition, other measures of body composition such as waist-to-hip-ratio were not available to examine the potential effect of central adiposity. The study population consists of men undergoing prostatectomy and may not reflect the full range of BMI that might be seen in the general population. Because the study population consists of men who underwent RP, the postoperative nomogram was used; therefore, the observed associations in the present study may not apply to other prostate cancer nomograms (e.g., pretreatment nomogram). The present study was a cross-sectional examination of obesity and tumor characteristics (and nomogram scores) measured at the time of surgery and therefore does not establish causality.

In conclusion, findings from the present study suggest that obese RP patients have a higher risk of experiencing prostate cancer progression. Identifying men at higher risk for treatment failure has the potential for better patient treatment decision making and may aid in the accrual for appropriate clinical trials. However, results of the present study need confirmation in other study populations such as large prospective cohorts with adequate length of follow-up before recommendations regarding treatment can be made or modifications to the nomogram tool are incorporated.
